# Innate Immunity Modulating Impurities and the Immunotoxicity of Nanobiotechnology-Based Drug Products

**DOI:** 10.3390/molecules26237308

**Published:** 2021-12-01

**Authors:** Claire K. Holley, Marina A. Dobrovolskaia

**Affiliations:** Nanotechnology Characterization Lab, Cancer Research Technology Program, Frederick National Laboratory for Cancer Research Sponsored by the National Cancer Institute, Frederick, MD 21702, USA; claire.holley@nih.gov

**Keywords:** immunity, bionanopharmaceuticals, impurities, immunotoxicity, immunogenicity, bioassays, nanomedicine

## Abstract

Innate immunity can be triggered by the presence of microbial antigens and other contaminants inadvertently introduced during the manufacture and purification of bionanopharmaceutical products. Activation of these innate immune responses, including cytokine secretion, complement, and immune cell activation, can result in unexpected and undesirable host immune responses. These innate modulators can also potentially stimulate the activation of adaptive immune responses, including the formation of anti-drug antibodies which can impact drug effectiveness. To prevent induction of these adverse responses, it is important to detect and quantify levels of these innate immunity modulating impurities (IIMIs) that may be present in drug products. However, while it is universally agreed that removal of IIMIs from drug products is crucial for patient safety and to prevent long-term immunogenicity, there is no single assay capable of directly detecting all potential IIMIs or indirectly quantifying downstream biomarkers. Additionally, there is a lack of agreement as to which of the many analytical assays currently employed should be standardized for general IIMI screening. Herein, we review the available literature to highlight cellular and molecular mechanisms underlying IIMI-mediated inflammation and its relevance to the safety and efficacy of pharmaceutical products. We further discuss methodologies used for direct and indirect IIMI identification and quantification.

## 1. Introduction

The body’s primary “innate” defense against foreign invaders is triggered by an immediate but relatively non-specific localized immune response including both cellular and biochemical components. The cells contain pathogen recognition receptors (PRRs) capable of tightly binding pathogen-associated molecular patterns (PAMPs) common to several classes of infectious agents [1]. PAMP binding by cognate PRRs triggers immune cell activation, chemokine/cytokine secretion, and biochemical mediators, including the complement system (both systemically produced by the liver and cellularly produced by the activated immune cells), ficolins, pentraxins, and the coagulation system. The coordinated function of these components leads to the hallmark signs of acute inflammation: redness due to increased blood flow and tissue permeability, swelling caused by increased leukocyte (neutrophil, basophil, monocyte) recruitment and subsequent fluid retention in affected tissues, heat (local), and fever (systemic) to decrease pathogen replication and activate production of complement proteins for pathogen opsonization, and pain from the previous effects which act as a warning to the host of tissue damage and infection [2,3]. Together, these processes work to destroy invaders as well as prevent and repair any further tissue damage.

Lastly, innate immune effectors promote the secondary “education” of the immune system against similar future attacks. For this, microbial antigens generated via pathogen phagocytosis are displayed on the surface of antigen-presenting cells (APCs), specifically macrophages and dendritic cells (DCs). Through co-stimulation by pro-inflammatory cytokines and APC-antigen presentation, T-cells differentiate into specialized subsets responsible for promoting enhanced B-cell activation (CD4^+^ helper T-cells), direct pathogen degradation (CD8^+^ cytotoxic T-cells), and immune modulation (regulatory T-cells (T_regs_)) [3,4]. Upon B-cell activation, gene rearrangement produces large quantities of highly variable and specific antibodies. While this “adaptive” immune response is slow compared to the innate immune response, these antibody-producing plasma cells are maintained long-term, the “memory” of which allows for more rapid recognition and a stronger, more specific immune response upon secondary antigen exposure [1].

Unlike the epigenetic recombination required by the adaptive immune response, trained immunity is a form of non-specific, T-cell independent innate immunity, which relies mainly upon macrophage activation and pro-inflammatory cytokine production for long-term functional reprogramming of innate immune cell responses. Therefore, secondary antigen exposure can lead to temporarily altered cellular responses, either enhanced or reduced, compared to the primary response [5]. Depending on the degree of “training,” protection can be conferred against reinfection by a specific microorganism and some additional non-specific protection against other unrelated pathogens [5].

To prevent inadvertent activation of these immune responses, new pharmaceutical compounds must go through several phases of investigation and regulatory review, consisting of discovery/development, preclinical testing, clinical testing, and approval, before being introduced to the market. Drug discovery/development encompasses the isolation (or fabrication) and subsequent characterization of a new compound, whether a molecule, nucleic acid sequence, or peptide/protein, for therapeutic use. This new compound is then subjected to preclinical (laboratory) testing, during which chemical or genetic analysis, pharmacological tools, and animal models are used to determine the safety and effectiveness of this drug towards a specific disease/condition. Due to the need for new drug compounds, half of all drug-related research and development expenditures occur during this stage, even though only one out of every thousand compounds progress to the next stage [6,7]. After successful testing in animal models, a new drug candidate is then deemed ready for clinical testing in humans. The clinical trial phases determine (I) the drug’s metabolic and pharmacological actions, side effects, and effective dosage in healthy patients; and then (II) the drug’s effectiveness in “diseased” patients as an improvement upon available treatments, if any. Of the compounds entering clinical trials, approximately 90% fail to pass the clinical phase I/II safety and efficacy requirements [7]. Those few compounds that do advance to clinical trial phase III are tested on a larger cohort of diseased patients to find the best balance between drug safety and effectiveness (dosage regimen, duration, etc.). Finally, once a therapeutic candidate has successfully passed these experimental hurdles, it must undergo final approval by a regulatory health agency (e.g., Food and Drug Administration (FDA) in the US) before being registered and sold as an available treatment [6]. Overall, from start to finish, the process of bringing a drug from the bench to the patient’s bedside can cost over USD 800 million and take 8–10 years of effort with no guarantee of final approval [6,8]. 

Due to the financial and societal costs of the extensive process required for drug development, testing, and approval, it is essential that any potential product “failure” not be the result of the inadvertent inclusion of innate immunity modulating impurities (IIMIs, a.k.a innate immune response modulating impurities, IIRMIs [9]), components of a biotherapeutic treatment other than the target product that can potentially trigger the development of an immune response in the recipient [9,10]. Herein, we review the available literature to highlight cellular and molecular mechanisms underlying IIMI-mediated inflammation and its relevance to the safety and efficacy of pharmaceutical products, and to discuss methodologies used for IIMI identification. Challenges with the detection and understanding of the immunotoxic effects of drug products arising from intrinsic immunological properties (e.g., immunosuppression, immunostimulation, immunomodulation, immunogenicity) of activating pharmaceutical ingredients (APIs) or intended formulation components (e.g., carriers and excipients) are not covered in this review as they have been extensively discussed elsewhere [11,12,13,14,15,16,17,18]. 

## 2. Innate Immunity Modulating Impurities

**Figure 1:**
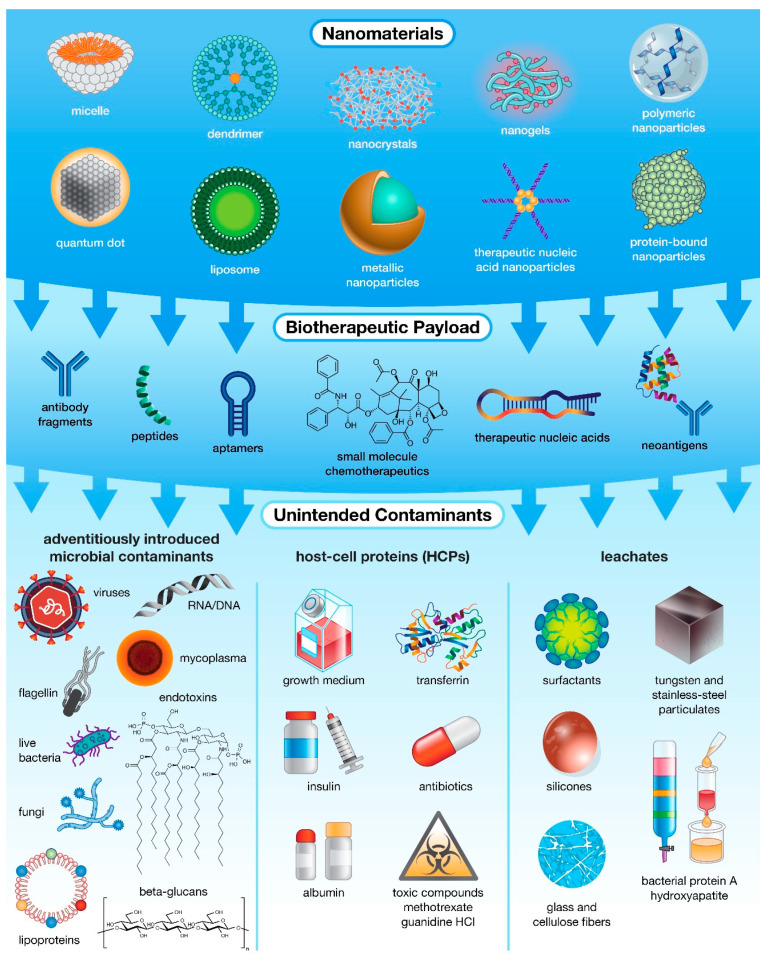
The many levels of possible unintended contamination in drug products. While most often associated with microbial contamination, unintended impurities can actually be introduced into pharmaceutical products from multiple sources, including raw materials and specialized host-cell reagents, and at various stages of production, ranging from fabrication and payload encapsulation in nanocarriers to purification of the final formulation.

IIMIs encompass everything from live microbial contamination and pathogen-derived antigens (proteins, sugars, nucleic acids) to compounds introduced during the nanobiotherapeutic manufacturing and purification processes (Figure 1) [19,20]. The first source of IIMIs is adventitiously introduced microbial contaminants including live bacteria, mycoplasma, fungi, viruses, or their by-products. While the most common source of these impurities is contaminated raw materials [10], other sources include non-sterile equipment, improper handling practices, or contaminated facilities, though these sources are less likely in a highly controlled facility that employs appropriate sterilization procedures [10]. The second source of IIMI contamination is from host-cell proteins (HCPs), proteins produced by modified host organisms that are unrelated to the intended recombinant product. The population of HCPs produced during biopharmaceutical manufacture depends on host cell type and strain, location of expressed product (cytoplasm, periplasm, external culture medium), physiochemical properties and modalities expressed by-product (charge, hydrophobicity, structure, post-translational modifications, etc.), and the techniques employed during recovery and purification [21]. Due to the limited subset of physicochemical properties optimized for purification, a sub-population of HCPs with similar attributes to the target product will normally co-purify regardless of the process employed [21]. In addition, the use of chemical additives needed to maintain these modified host cells (e.g., growth medium, transferrin, albumin, insulin), as well as chemical additives and selective pressure agents applied for increased product production and modification (e.g., methotrexate, antibiotics, guanidine HCl) can result in adverse patient reactions and can potentially lead to the formation of antibiotic resistant bacterial strains [10]. Lastly, even processes employed for product filtration and purification can inadvertently introduce impurities that leach into the final product. Common “leachates” include bacterial protein A which is normally used for isolating antibodies, as well as hydroxyapatite, tungsten and stainless-steel fragments, glass and cellulose fibers, surfactants, and silicones which can be introduced by filters or containers used during the manufacture and purification processes [10,22]. Ideally, detection of such “leachates” in a biopharmaceutic product will result in modification and/or augmentation of purification processes, such as the use of high-quality resins, to prevent introducing these impurities [10]. Overall, at each stage biopharmaceutical production, there is the potential to introduce IIMIs which may have little/no impact on the function of the resulting drug product but are potent immune activators that have the potential to trigger an undesirable host immune response [23].

**Figure 2:**
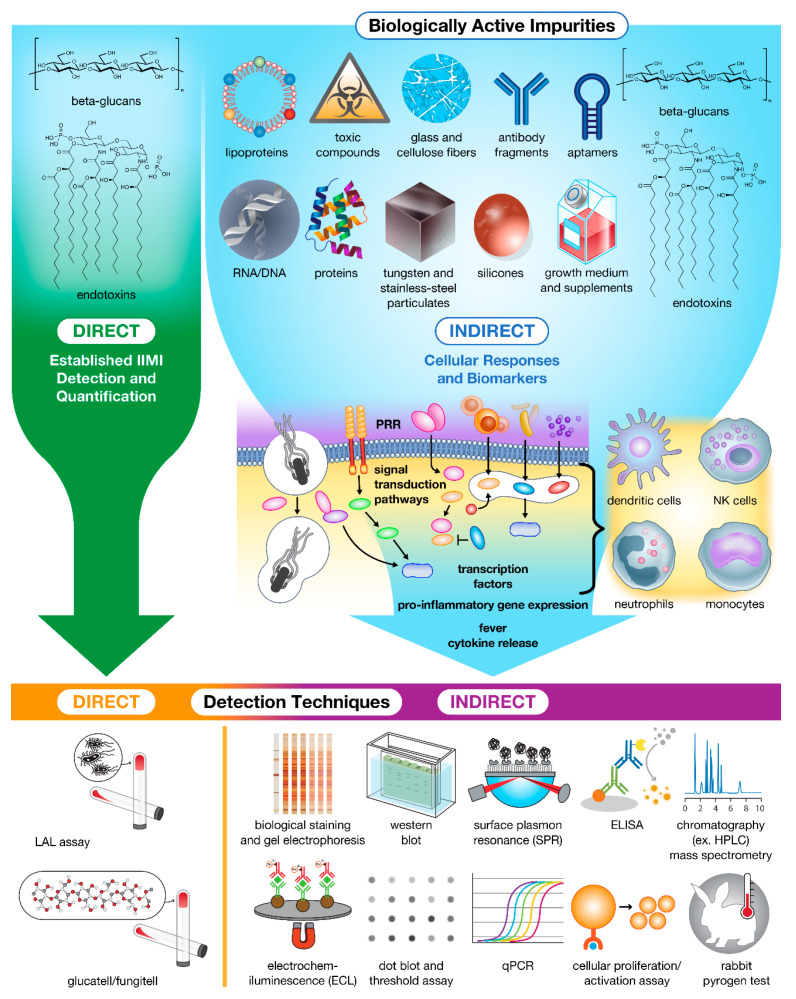
Impurities in drug products trigger innate cellular responses and produce biomarkers for bioassay detection and Quantification. Currently, only β-glucans and endotoxins can be detected and quantified directly using specialized assays. The remaining population of impurities must instead be detected and quantified indirectly using downstream biomarkers (e.g., proteins, peptides, and nucleic acids) and immune cell activation as hallmarks of contamination.

When in the presence of these IIMIs (Figure 2), immune cells (e.g., DCs, macrophages, monocytes, neutrophils, and some epithelial cells) recognize these antigens via a variety of pattern recognition receptors (PRRs) containing leucine-rich repeats (LRR) [24,25], including toll-like receptors (TLRs), nod-like receptors (NLRs), retinoic acid-inducible gene-I (RIG)-I-like receptors (RLRs), and C-type lectin receptors (CLRs). Each of these receptor families binds highly conserved microbial structures containing pathogen-associated molecular patterns (PAMPs), or endogenous structures containing damage-associated molecular patterns (DAMPs) released via cell rupture which are important for augmenting the elimination of pathogens and pathogen-damaged cells [9,26,27].

The most studied and diverse family of PRRs, TLRs are a family of highly varied signaling receptors, each of which binds to a different set of microbial structures to trigger intracellular signaling resulting in cytokine secretion and lymphocyte activation [24,26]. Membrane-tethered TLRs, which often require dimerization for appropriate antigen binding and subsequent intracellular signaling, bind to molecules found on bacterial surfaces, including triacyl lipopeptides/proteins, glycolipids, and peptidoglycans, all of which bind to either the TLR1/2 heterodimer or the Dectin1/TLR2 heterodimer; diacyl lipopeptides, lipoteichoic acid, or zymosan which bind to TLR2/6; lipopolysaccharides (LPS) or endotoxins, which bind to MD2, an extracellular adaptor protein for TLR4; and flagellin, which binds TLR5 [24,25,28]. Several DAMPs can also bind membrane-tethered TLRs, including but not limited to hyaluronic acid and other fatty acids, high-mobility group protein B1 (HMGB1), heat shock proteins, S100 proteins, fibrinogen, and tenascin-C which bind to TLR4 [29,30] and serum amyloid A protein, which binds the TLR2/6 heterodimer [27,31]. On the other hand, intracellular TLRs bind to microbial components released after pathogen endocytosis and phagocytosis, including viral double-stranded (ds) RNA containing poly(I:C) motifs which binds TLR3; unmethylated CpG-rich DNA which binds to TLR9; and Guanosine/Uridine-rich single-stranded (ss) RNA and anti-viral imidazoquinoline compounds that mainly bind to TLR8 but can also bind TLR7 [24,25,27,28] Many intracellular TLRs also recognize DAMPs. For example, TLR7 and TLR9 distinguish between snRNP immunocomplexes vs. immunocomplexes of self-DNA or histones respectively [27].

With the assistance of a variety of signaling adaptor proteins (TIRAP, TRAM) and TRIF/TRAF transcription factors [24,25], all antigen-bound TLRs, except TLR3, activate intracellular signaling through a myeloid differentiation primary response protein (MyD88)-dependent NFκB pathway resulting in the secretion of pro-inflammatory cytokines, including type II interferons (IFNs) (e.g., IFNγ), interleukins (ILs) (e.g., IL-1β, IL-6, CXCL8/IL-8, IL-12, and IL-18) and tumor necrosis factor α (TNFα); priming of caspase-1; and the activation of local lymphocytes and vascular endothelium, eventually resulting in antibody production [24,26]. Meanwhile, MyD88-independent activation of IRF3/7 leads to the type I IFNs (IFNα) response critical for antiviral defense [25,30]. However, the continuous stimulation of these PRRs, especially the “bipolar” PRRs involved in DAMP recognition, can lead to inflammatory dysregulation leading to the development of autoimmune and chronic inflammatory diseases [27], as well as blunted responses, also known as tolerance [32]. As such, these pathways are tightly controlled, with some TLRs (TLR2 and TLR4) even having decoy receptors designed to dampen innate responses during severe infection by blocking the interactions between the bacterial ligands and the active TLRs [26].

TLR function also overlaps and integrates with other PRR signaling pathways, including NLRs, RLRs, and CLRs. NLRs, such as NOD1 and NOD2, act as intracellular bacterial sensors by recognizing peptidoglycans (e.g., mDAP and MDP respectively) resulting in inflammasome-mediated NFκB activation leading to the production of IL-1β [26]. The TLR and NLR pathways are clearly integrated for producing IL-1β, as effective NLR activation requires both PAMP activation of the inflammasome and TLR priming, to initiate an inflammatory response [26]. Other NLRs are responsible for triggering the activation and regulation of pro-inflammatory caspase-1 and caspase-5. RLRs, on the other hand, are intracellular viral sensors, binding specifically to dsRNA. Like NLRs, these receptors contain caspase-recruitment domains (CARD) responsible for recruiting adaptor proteins resulting in IRF3 and NFκB activation, leading to the production of type I IFNs (IFNα/β) and pro-inflammatory cytokines (e.g., TNFα, IL-1β, IL-6). Due to these similarities with viral-sensing TLRs (i.e., TLR3, 7, 8, and 9), it is likely that TLRs and RLRs also function together to provide ubiquitous anti-viral protection [26]. Lastly, CLRs are carbohydrate-binding receptors located mainly on the surface of DCs [33,34]. Group I CLRs, which bind mannose and fucose, aid in pathogen phagocytosis, degradation, and antigen presentation to T-cells [33]. Group II CLRs, which bind glucan and dectin, appear to be more immunomodulatory; they induce upregulation of IL-10 and the secretion of cytokines (specifically IL-1β, IL-6, IL-12, and IL-13) required for T-cell polarization into the T_H_1 or T_H_17 subsets [33,34]. CLRs also act in collaboration with other TLRs (TLR2, 4, 5, 7, and 9) to amplify preceding TLR-mediated NFκB activation and cytokine induction, in addition to triggering the complement cascade through β-1,3-glucan binding complement receptor-3 (CR3, CD11b/CD18), located in the membrane of many phagocytic cells [24,33,34]. 

Overall, while the binding domains and adaptor proteins vary, there is a significant overlap between the downstream signaling domains employed by each of these pathways. However, these pathways are far from redundant. While TLR7 and TLR9 are expressed on the endosomes of many cells including DCs, eosinophils, basophils, and B-cells, TLR3 and TLR8 are only expressed by natural killer (NK) cells [24]. In the same way, where TLRs are located mainly on leukocytes (macrophages, DCs, neutrophils, etc.), NLRs and RLRs can be found on all cells except DCs [26]. The complex signaling interplay between these pathways, in response to bacterial and viral antigens, highlights the importance of pro-inflammatory cytokines and PAMP-PRR detection in providing a tailored front-line defense against a wide variety of invading pathogens [26]. Further, the interplay between these PRR signaling pathways also drives the induction of effective adaptive immune responses, in that IL-1R and caspase-1 play a crucial role in development of both CD4^+^ and CD8^+^ T-cells, as well as antibody responses [25]. As such, IIMI-induced immune responses in the presence of biological therapeutics can lead to immunogenicity toward the administered biologic and potentially to other similar endogenous proteins [19], which can result in loss of treatment efficacy as well as severe and potentially lethal clinical consequences including anaphylaxis, serum sickness, and the formation of autoimmunity [19].

## 3. Impact of IIMIs on the Immunotoxicity of Drug Products

In the presence of IIMIs, activated phagocytes secrete both stimulatory and inhibitory cytokines to drive and regulate the immune response (Figure 2). These small proteins, which include interferons (IFNs), interleukins (ILs), tissue necrosis factors (TNFs), and chemokines, create a multilevel signaling network that elicits inflammatory responses, angiogenesis, as well as cellular activation, proliferation, and differentiation. IFNs play a central role in innate immunity to viruses and other microbial pathogens [2,29]. ILs function mainly as immune system regulators, responsible for immune cell differentiation and activation [2,29]. Multifunctional TNFs activate vascular endothelium permeability to allow entry of complement proteins and effector cells; increase fluid drainage to lymph nodes to clear pathogens and educate T/B-cells; and stimulate the production of IL-6 responsible for systemic fever, metabolite mobilization, and shock [2,29]. As the largest family of cytokines, chemokines have many diverse functions, ranging from controlling cell migration (e.g., recruitment and activation of local neutrophils and basophils to the site of infection), to such diverse processes as embryogenesis, innate and adaptive immune system development and function, and cancer metastasis [2,3].

Under normal circumstances, cytokine-driven immunostimulation is protective, such as when it is triggered by adjuvants to increase vaccine potency. However, when immune stimulation is unexpected or uncontrolled, especially in the presence of therapeutic compounds, it leads to unintended cellular immune responses and/or antibody production in response to that drug product. Such immunotoxicity encompasses ‘any adverse effect on the structure or function of the immune system, or other systems affected by the same biological mediators (e.g., nervous and endocrine systems), as a result of immune system dysfunction’ [35]. Immunotoxicity is further classified by the level of response, including (1) non-specific immunostimulation, (2) uncontrolled hypersensitivity (allergy, autoimmunity, and chronic inflammation) leading to tissue damage, and (3) immunosuppression [35].

In the most general terms, immunostimulation is the normal, controlled activation of an immune response (“sensitivity”) to an antigen, an important prerequisite for immunogenicity [36,37]. Weak antigen sensitivity responses due to the simple presence of an antigen often fail to elicit sufficient immune activation required to trigger humoral or cellular immunity and subsequent clinical effects [36]; whereas moderate immunostimulatory responses, which might require the assistance of an adjuvant for additional phagocyte activation and cytokine secretion, can result in the eventual downstream production of neutralizing antibodies leading to therapeutic immunogenicity [22,38]. The most common symptoms of immunostimulatory reactions are fever, chills, malaise, hypotension, and localized tissue inflammation (redness, heat, swelling, and pain) around application [2,3,39]. These symptoms are often quickly resolved or can be controlled through the application of immunosuppressive agents such as recombinant chemokines or monoclonal antibodies [36].

Inappropriate or inadequately controlled immunostimulation may lead to hypersensitivity reactions (HSRs) [37]. While no universal classification of HSRs exists, the system proposed by Gell and Coombs, which classifies HSR reactions based on underlying mechanisms, time of symptom occurrence, mediators, and clinical manifestations, is frequently used [40]. Type I HSRs, or classic “acute allergic” reactions such as asthma or food allergies, result from antigen binding to immunoglobulin E (IgE) antibodies on the surface of granulocytes (basophils, mast cells), triggering cellular degranulation and an immediate release of histamine, leukotrienes, and other mediators [40,41,42]. While also antibody-driven, type II HSRs lead to the production of IgM and IgG antibodies as well as the activation of complement, natural killer (NK) cells, neutrophils, and macrophages [41], all of which result in cellular cytotoxicity and tissue damage. These types are reactions are commonly seen in response to medications such as penicillin, thiazides, or cephalosporins. Type III HSRs are driven by uncontrolled systemic complement activation, resulting in large deposits of IgM immuno-complexes and anaphylatoxins C3a and C5a in tissues which can trigger cell death and compromise organ function [40,43]. Examples of this type of HSR include serum sickness and autoimmune diseases such as rheumatoid arthritis and lupus erythematosus [42]. As both type I and type III HSRs result in the degranulation of basophils and mast cells, true IgE-mediated type I allergy reactions, which are referred to as “anaphylaxis” even though they lack complement involvement, are often difficult to distinguish from IgE-independent complement-activation related pseudoallergy (CARPA) reactions, also known as anaphylactoid or pseudoallergy, which do rely on complement anaphylatoxins C3a and C5a [42,44,45,46]. Lastly, type IV HSRs such as contact dermatitis or drug sensitivities, are delayed T-cell and macrophage-mediated reactions characterized by increased cytokine release and lymphocyte stimulation [40,42].

Anaphylatoxins and activation of immune cells by PAMPs and DAMPs, also trigger cytokine responses. Since cytokines are pleiotropic and have overlapping functions, they are normally very effective for small-scale localized responses [3]; however, whatever the antigenic trigger, the unregulated overproduction of cytokines due to strong/hyper-immunostimulation (a.k.a cytokine storm or cytokine-response syndrome) can quickly spread unchecked throughout the body via the circulation, resulting in overwhelming systemic inflammation, catastrophic tissue damage, disseminated intravascular coagulation (DIC), and death [2,22,24,38]. Due to their systemic nature, cytokine storms are most often associated with severe, widespread infections, high levels of IIMI contamination (e.g., endotoxins at doses above 5 EU/kg), or massive tissue damage (e.g., shock/trauma) [2,47,48,49].

Cases of delayed unregulated cytokine secretion coupled with prolonged tissue infiltration by activated macrophages and lymphocytes can also lead to other serious immunological consequences, such as the formation of chronic inflammatory or autoimmune diseases [35,39]. While differentiated by the source of the inflammatory trigger, either endogenous (autoimmune) or exogenous (chronic inflammatory), the general result is the same. Excess TNF production is associated with a number of chronic inflammatory and autoimmune diseases [2,29] while an over-activation of the complement system has been implicated in the pathophysiology of asthma and acute respiratory distress syndrome [43]. Similarly, prolonged exposure to over-activated immune cells, cytokines, and antibody/immune complexes can trigger the formation of granulomas, a common defense mechanism in which harmful components are isolated away from healthy tissue. These chronic HSRs are debilitating as well as life-threatening, since the cells of the immune system are continuously attacking healthy tissues resulting in chronic pain, injury, and eventually organ failure [35].

Lastly, effective immune responses are normally a delicate and tightly controlled balance between stimulation and suppression. The systemic production of IL-10 is associated with the downregulation of neutrophil and monocyte function, working as an anti-inflammatory response following systemic inflammation [2,29]. While this natural counterbalance is conceptually beneficial in controlling systemic responses to local infections, immunotoxicity can occur when immunosuppression or dysregulation leads to an inappropriately reduced immune response resulting in frequent and serious adverse effects [35]. Since the majority of destructive immune responses are associated with HSRs, as previously discussed, many immunosuppressive therapeutics attempts to dampen overactive pro-inflammatory responses but instead have been reported to exacerbate asthma, eczema, and psoriatic lesions [2,39]. Dampening/deficiency of normal immune functions, such as the inhibition of T-cell function and adaptive immune responses, has also been associated with more frequent opportunistic concomitant infections (e.g., pneumonia, Candida, Kaposi’s sarcoma, etc.) [35].

After activation by pro-inflammatory cytokines and PRR binding, local antigen-presenting cells (APCs), such as macrophages and dendritic cells (DCs), endocytose and degrade invading pathogens. APCs present fragments of these degraded pathogens on their membrane-bound major histocompatibility complex (MHC) receptors, which bind to and activate T-cells, initiating their downstream activation of B-cells [3,4,24,33]. The fate of activated T-cells is determined by the levels and types of cytokines induced during the inflammatory response, as well as the type and dose of antigen, type and affinity of MHC binding, route of administration, presence of other adjuvants, and patient genetic predisposition [4]. Major classes of T-cells include CD4^+^ helper (T_H_) T-cells activated by MHC class II antigen presentation, CD8^+^ cytotoxic T-cells activated by MHC class I presentation, and regulatory T-cells (T_regs_) [3,4,33]. In the presence of either IFNγ or a combination of IL-4, IL-6, and PGE-2, naive CD4^+^ helper T-cells are further differentiated into specialized subsets of CD4^+^ helper T-cells which are responsible for cell-mediated (T_H_1) or humoral (T_H_2) responses respectively [3,4]. T_H_1 T-cells secrete large quantities of IFNγ, in addition to IL-2, IL-3, IL-12, IL-18, GM-CS, and TNFβ, to regulate the inflammatory response and fight intracellular pathogens and viruses [3,4,33]. These cytokines promote macrophage activation and the production of opsonizing and complement-fixing antibodies. However, if not properly regulated, T_H_1-dependent immune reactions can also lead to antibody-dependent cellular toxicity and delayed HSRs, the most predominant of which can include autoimmune disorders, acute allograft rejection, and chronic inflammatory disorders [2,4,39]. On the other hand, T_H_2 T-cells secrete large quantities of IL-4, IL-5, IL-13, in addition to IL-3, IL-6, IL-9, IL-10, GM-CSF, and TNF, to induce humoral responses and mucosal immunity, as well as fight helminths and extracellular pathogens [3,33]. These cytokines promote the proliferation of mast cells and eosinophils, favor the differentiation of IgE and IgG-producing B-cells, and facilitate the synthesis of mucosal IgA [3,4]. While T_H_2 cells predominate in transplantation tolerance, they can also lead to chronic graft vs. host disease, systemic sclerosis, and allergen-reactive atopic disorders [4,39,43]. 

While it has been observed that cytokines from specific T_H_ cell subsets (e.g. IFNγ from T_H_1 cells and IL-10 from T_H_2 cells) usually inhibit the action of the other types of T-cells and their companion phagocytes [3,4], this classic binary model does not account for instances where an immunological response is triggered without any significant shift in T_H_1/T_H_2 balance, such as is the case with omega-3 fatty acids, or alternatively where there is T_H_1/T_H_2 activation with minimal immunological pathogenesis, such as with melanin, probiotics and zinc [3]. In addition, other sub-classes of T-cells have been identified which were not previously represented by this model, including but not limited to: T_H_17 cells, which secrete IL-17 to mobilize phagocytes against extracellular fungi and bacteria; and T_regs_, which produce FoxP3 to control the activity of the other effector T_H_ cells and maintain immunological tolerance to self-antigens [3,19,23,33]. However, increased levels of regulatory (T_H_17, T_reg_) cytokines such as IL-10 or IL-17 can also be an indication of adverse patient effects such as autoimmune diseases or advantageous concomitant infections [3].

## 4. Sources of Immunotoxicity in Nanotechnology-Based Products

The use of nanoscale platforms (e.g., dendrimers, liposomes, nanoparticles, nanotubes, nanogels, etc.) has become a popular technique to reduce drug immunotoxicity while improving therapeutic solubility, biodistribution, and cell-specific delivery compared to the traditionally formulated versions of these drugs. However, it has been noted that some nanocarriers can themselves be immunomodulatory (Figure 1), such as RNA nanoparticles which have been shown to induce pro-inflammatory cytokine secretion and enhance inflammation [11,50]. The raw materials used for nano-platform fabrication can have various immunological effects, either due to previously discussed contamination or due to the chemical properties of the material itself. Some nanomaterials are immunostimulatory, such as lipid-based nanocarriers and carbon nanotubes which have been shown to induce cytokine production and inflammation [50,51,52], while other nanomaterials are immunosuppressive including PEGylated NPs which lead to TLR9 inhibition and immune cell avoidance [50,51,53]. Similarly, the processes employed during nanocarrier synthesis and purification often use immunotoxic reagents, such as surfactants such as cetyltrimethylammonium bromide (CTAB); peptizing agents such as polystyrene sulfonate (PSS); or complexing agents such as nickel, to improve drug loading or enable molecule crosslinking [54]. While these chemicals are not generally intended to be in the final product, trace elements (“leachates”) that remain after washing and filtration can induce cytokine production and inflammation, compounding the other immunomodulatory aspects of the nanocarrier [54]. 

Once fabricated, the physical properties of the nano-formulation, including size, shape, and surface charge, can also alter immunotoxicity. Nanoparticle interactions with the immune system have been extensively discussed elsewhere [11,12,13,50,55,56,57,58]. Here, we will use some examples to demonstrate structure–activity relationship between nanoparticle physicochemical characteristics and their immunological properties. First, several studies have shown that smaller particles (<500 nm) promote humoral T_H_2 responses, compared to very large particles (>1 μm) which have been found to stimulate cell-mediated T_H_1 responses. In addition, very small particles (<100 nm) are associated with increased CD8^+^ and CD4^+^ T-cell activation compared to their larger (>500 nm) counterparts, who induce good antibody responses [59]. Thus, small particles may invoke virus-like responses and larger particles induce bacteria-like responses [59]. Second, compared to spherical nanocarriers, oval-shaped liposomes and carbon nanotubes have been shown to activate complement and platelet aggregation with membrane rupture, respectively [50,60]. Finally, cationic carriers are more immunostimulatory than anionic or neutral carriers, triggering cytokine secretion (TNF, IL-12, IFNγ); activation of DCs, T-cells, and neutrophils; and procoagulant leukocyte and platelet activation which can potentially lead to DIC [12,50,61]. Taken together, while a nanocarrier is often designed to reduce the immunotoxicity of a therapeutic payload, the chemical and physical properties of that nanocarrier along with it being a source of undesirable IIMIs contamination may lead to an exaggeration of the immunotoxicity of the final drug product. For example, cationic polyamidoamine (PAMAM) dendrimers in the presence of low amounts of endotoxin have a variety of immunotoxic effects that neither dendrimers nor low levels of endotoxin alone have [11,50]. Therefore, the use of nanomaterial platforms should be considered as yet another source of IIMIs. Translational and regulatory challenges arising from immunomodulatory properties of nanocarriers and their ability to exaggerate immunotoxicity of low levels of IIMIs (e.g., endotoxin) have been extensively discussed elsewhere [12,13,50,56,61]. Immunogenicity of nanoparticles alone and in the context of IIMIs along with nanoparticle contribution to the immunogenicity of protein-based therapeutics have also been reviewed earlier [22].

## 5. IIMIs Commonly Found in Pharmaceutical Products

### 5.1. Microbial Components

When it comes to assessing biotherapeutic purity, the only current consensus is that it is important for manufacturers to minimize the potential for their formulation to trigger adverse patient reactions and future immunogenicity by removal of microbial or host cell-related impurities, as summarized by testing standards (Table 1) [29,35,62,63,64,65,66,67,68]. Currently only a fraction of the potential IIMIs, specifically lipopolysaccharide (LPS), β−glucan, flagellin, HMGB1, and nucleic acids, are routinely measured during immunotoxicity screening of biotherapeutics [69] to confirm that the levels of these IIMIs fall within the FDA-approved 1–100 ppm range [21]. In addition, due to the breadth and complexity of potential IIMIs, there is currently no single assay that can provide a profile of all IIMIs present within a biotherapeutic [70]. Other than the fact that any assays used to detect IIMIs and evaluate possible immunotoxicity should be tailored to the specific contaminant [62], there is currently very little agreement as to which analytical assays should be standardized for IIMI screening [21]. Therefore, most studies use a series of assays to broadly cover the detection of all possible IIMIs present in biopharmaceuticals [21,71], including single analyte mechanistic assays, basic staining/gel-based assays, immunoassays, and cellular-based assays (Figure 2).

### 5.2. Whole Microbes 

After biopharmaceutical manufacture and microbial inactivation via low pH adjustment, heat, and solvent/detergent treatments [10], filtration is used for the removal of bulk impurities such as neutralized pathogens (bacteria, viruses), destabilized protein aggregates, or other bulk contaminants [10,22]. Due to the comparatively large size of these impurities, microscopy techniques such as transmission electron microscopy (TEM) [10], have been used to assess the effectiveness of these initial filtration steps. These high-resolution microscopy techniques employ lasers or electrons beams and extensive sample preparation to achieve a 0.1–1 mm visualization limit [73,74], which makes them time- and cost-prohibitive. Further, given their inability to provide accurate IIMI quantification, microscopy techniques such as TEM can only provide an indication as to what additional filtration and purification steps may be required; as these filtration techniques may not be sufficient to completely remove all traces of IIMIs, more accurate IIMI detection and quantification must then employ antigen-specific assays [31,73].

### 5.3. Leachates

After filtration, a range of chromatographic techniques are used for drug concentration and purification, to remove impurities such as drug by-products, unprocessed raw materials, and other leachates that may have been introduced into the formulation during the manufacturing process [10,22]. For complete sample separation, chromatography exploits the physical characteristics of the target protein/peptide in solution, including size, mass, ionic charge, binding affinity, pH, and electrokinetics, to partition it away from other components that may be present in the solution after fabrication [75]. Some of the chromatography techniques previously used for assessing biotherapeutic purity include ion exchange, size exclusion, capillary electrophoresis (CE), micellar electrokinetic chromatography (MEKC), and reverse-phase high-performance liquid chromatography (HPLC) [75]. Often referred to as “high pressure” liquid chromatography due to how the sample in the mobile phase is pressurized before injection into the absorbent stationary phase column, HPLC has become one of the most popular chromatography methods due to its high-performance detection, separation, and quantification of very small volumes (5–50 μL) of samples including degradation by-products, IIMIs, and unprocessed raw materials. HPLC is often used to separate molecules that are not large enough or charged enough for adequate separation by traditional size-exclusion chromatography or ion exchange-chromatography respectively [75]. While separation efficiency and quantification analysis are highly accurate, this technique requires extensive protocol optimization for the best results [75] in addition to specialized equipment and a trained operator. Additionally, chromatography can typically only separate one IIMI at a time, though multidimensional chromatographic separations paired with fluorescence detection are currently being pursued [71]. 

Sub-visible particles, which can include anything from small molecules to the components of protein aggregates, can also be identified using mass spectrometry (MS) techniques [21,76]. MS separates charged molecules or fragments by accelerating them through an electric or magnetic field, which separates the molecules based on their mass-to-charge ratio and then identifies them by correlation with known molecule masses and fragmentation patterns. This technique is especially important in identifying the relative concentrations of impurities and degradation products relative to target drug products during pharmaceutical development [77]. As a pivotal technique in the process of molecule structure elucidation [77], high-resolution MS/MS is now also being used to identify and quantify larger, more complex impurities and proteins that can be isolated from the bands of an electrophoresis gel or sampled directly from solution using liquid chromatography-tandem mass spectrometry (LC-MS/MS) [20,21]. Due to improvements in high-throughput capabilities combined with improved sample preparation (e.g., chromatography fractionation and 2D gel electrophoresis), LC-MS/MS is now also being used for complete proteomic characterization and identification of complex therapeutic samples [21]. MS analysis is more precise than immunoassays but requires specialized equipment and analysis software, as well as trained personnel [76].

### 5.4. Host Cell Proteins

The most difficult IIMIs to isolate and quantify are host-cell proteins (HCPs) due to the diversity and complexity of the potential protein repertoire, as well as HCP similarities to the target drug product [69]. As there is currently no single assay that can detect and quantify all possible HCP-based IIMIs within a biotherapeutic formulation [70] nor any absolute control limits required by pharmaceutical regulators [21], most quality assurance uses a combination of methodologies to confirm drug product purity. A typical strategy often includes generic IIMI clearance studies such as the *Limulus* amebocyte lysate (LAL) test or mass spectrometry; sensitive silver staining (and immunoblotting) of electrophoretic gels; and quantitative HCP-specific immunoassays such as ELISAs [71], all of which will be discussed below.

## 6. Immune-Mediated Adverse Effects to Pharmaceutical Products

The combination of a strong immunostimulatory response [3,35,43] and the activation of specialized subsets of T-cells leads to target-specific destruction of pathogens and cancer cells, either by direct interaction with CD8^+^ T-cells and natural killer (NK) cells or by CD4^+^ T-cell activation and proliferation of B-cells to produce antigen-specific antibodies [19,23,24,78]. This IIMI-driven immunogenicity can lead to the formation of antibodies of different isotypes (e.g., IgM vs. IgG vs. IgE), allotypes (e.g., reflecting genetic differences between IgG of biologically unrelated individuals), and idiotypes (e.g., reflecting binding to specific epitopes within antibody variable sites) [19,23,79,80,81], resulting in anti-drug antibodies (ADAs) with varying impacts on drug effectiveness. Binding antibodies attach to a non-active portion of the therapeutic and therefore have little/no effect on therapeutic function, whereas cross-reactive neutralizing antibodies bind to therapeutic active sites, thereby neutralizing therapeutic function while also binding similar endogenous proteins and breaking immunological tolerance [19,23,82,83,84]. The presence of these ADAs can also have different functional consequences to the host including the HSR/anaphylaxis and autoimmune responses previously discussed [19,23,35,79,80,81]. The relationship between the occurrence of a specific antibody type and the impact on the patient are inversely related; binding antibodies are the most common but have the lowest clinical impact, while cross-reacting neutralizing antibodies are rare but have the highest clinical impact [23,79,80,81,85]. Therefore, it is important to understand, measure, and prevent this response from being induced. 

During the fabrication and production of drug compounds, there are many potential sources for the introduction of IIMIs into the final biotherapeutic formulation (Figure 1) [19,20]. In addition to the impurities/contaminants previously discussed, there are also several product-related and host-related factors that may have little/no impact on the function of the resulting drug product but have been shown to impact the immunotoxicity and immunogenicity of biotherapeutics [19,23,78]. Product-related factors include structural properties of the drug (sequence, epitopes, post-translational modifications), exposure to antigenic sites, solubility, formulation stability and storage, downstream processing, presence of impurities/contaminants that might be introduced during processing [19,78]. These factors can be mostly controlled through careful optimization and modification of the fabrication/purification processes. Further compounding the risk of immunogenicity are host-related factors, including host genetic predisposition, endogenous protein genetic variants, concomitant illnesses (e.g., kidney or liver diseases), host immune status (e.g., autoimmunity, prior exposure) as well as the treatment dose, duration, and route of administration [19,23,78]. 

## 7. Methods for IIMI Detection

### 7.1. Direct Detection Methods

The first bioassay used to measure the presence of bacterial contamination was the rabbit pyrogen test (RPT) which detected pyrogens, any contaminant that induces a histamine response, fever, chills, and other unwanted inflammatory side effects. The rabbit pyrogen test detects all pyrogens, so it is subject to high variability and low selectivity, in addition to being expensive and requiring extensive use of animals [10,31]. As an improvement, the *Limulus* amebocyte lysate (LAL) test detects the hemolymph coagulation of the American horseshoe crab *Limulus polyphemus* when in the presence of bacterial endotoxin/LPS and is used as a standard for bacterial contamination [86,87]. However, this assay is specific for endotoxin, not general pyrogens [31], and has reduced specificity in the presence of fungal β-glucans because the horseshoe crab lysate used for this assay contains two proteins that trigger activation of the proteolytic cascade: factor C is specific to the presence of endotoxin while factor G is specific to β-glucans [88,89]. Knowing this, a modified version of the LAL assay containing glucan-blocking reagents or recombinant factor C overcomes β-glucan interference during endotoxin detection [90]. 

While β-(1,3)-d-glucans are not as immunologically potent as bacterial endotoxins, requiring μg/mL concentrations as compared to the endotoxin pg/mL concentrations to elicit an immunomodulatory response, they are a common IIMI present in many pharmaceutical products and solutions [89]. Moreover, while there is currently no compendial standard for β-glucan detection or acceptable levels, a modified version of the LAL assay is growing in popularity [90]. Since LAL factor G is specific to β-glucans, factor C depletion from the LAL lysate enhances the assay’s sensitivity solely to β-glucan detection [89]. It is important to note that β-glucans are naturally introduced in a person’s diet, so data generated from β-glucan quantification assays need to be from clinically relevant doses of the drug formulation [89].

Challenges with endotoxin and beta-glucan detection in nanoformulations stemming from carrier-, excipient-, or drug-mediated interferences, mechanisms of interferences, and ways for overcoming them have been identified and extensively discussed earlier [11,89,91,92,93,94,95]. 

### 7.2. Indirect Detection Methods

For the development of effective assays, an appropriate biomarker can consist of any compound (e.g., metals, solvents, pathogens, etc.) or useful characteristic, such as a mechanistic by-product, which can be measured or evaluated, either directly or indirectly, and used as an indicator of normal biological, pathogenic, or pharmacologic processes [83,84]. Therefore, any of the product- or host-related impurities previously discussed, as well as raw materials used during the product’s manufacture and purification, can technically be considered a potential biomarker [85]. During method development, quantitative assays must be validated using appropriate controls and quantification must employ a standard curve of known analyte concentrations to determine the range of conditions under which appropriate levels of confidence can be attributed to the reproducibility and accuracy of the data [84,96]. Further, the validated assay must then demonstrate both sensitivity and specificity for the biomarker [84], such that the biomarker is correctly identified (i.e., true positive, sensitivity) at clinically relevant (ng/mL to pg/mL) concentrations [96] without also reacting to residual therapeutics or other impurities likely to be present within the therapeutic formulation (i.e., true negative, specificity). Reduced sensitivity can result in mistakenly missing the presence of IIMIs in a formulation (i.e., false negative) resulting in possible dangerous clinical manifestations and immunogenicity, while reduced specificity can result in misidentification of inert compounds as IIMI (i.e., false positive) leading to incorrect quantification and product disposal rather than administration to patients. Overall, when balancing these two parameters, increased sensitivity is often preferred to increased specificity.

### 7.3. Biological Staining and Gel-Based Methods

Biological staining is a common technique to detect and visualize the presence of HCPs and other impurities. This technique utilizes Coomassie Blue or silver staining to highlight the presence of protein analyzed by multidimensional (2D or 3D) gel electrophoresis [71] or fixed in histological samples, respectively [21]. While the sensitivity of these staining techniques is quite high, selectivity is not; this technique cannot discriminate between types or sources of proteins so other techniques need to be employed to further identify and quantify the protein contaminants [10]. Newer versions of this method employ fluorescent stains, such as SyproRuby, for 10–100 times increased sensitivity compared to previous stains since these stains are not dependent upon the protein composition [21]. Other stains also have improved specificity by binding to specific cellular elements (i.e., nucleic acids, carbohydrates, chromatin, etc.) though this method is still largely qualitative [21]. Gel electrophoresis and protein staining have progressed to the use of the more quantitative Western blot, a common antibody-dependent detection method [21] that has merit for identifying low (pg/mL) concentrations of protein impurities. Contaminating HCPs and product-related impurities are separated from the target biologic by gel electrophoresis [10], and then transferred to a PVDF or nitrocellulose membrane. Primary antibodies raised against HCPs are incubated with the membrane to allow for the formation of antigen–antibody complexes, which are then detected through secondary enzymatic or fluorescent labeling [21]. While this technique is both sensitive and specific, it requires the use of separate polyclonal antibodies against each impurity for optimal detection, which can be time and cost prohibitive in the long run [10]. In addition, this technique needs to be supplemented with additional immunoassays to help distinguish between process- or product-related impurities and impurities that might comigrate with the product [10]. 

### 7.4. Antibody-Based Enzymatic Methods

Surface plasmon resonance (SPR) uses antigen-ligand binding on a sensor chip to generate a signal due to a change in the refractive index caused by a difference in mass as the analyte binds to the ligand. Most often used to detect the presence of antibodies rather than antigens, this assay is capable of continuous measurements of binding interactions in ‘real-time’ [84]. For the detection of immunotoxic antigens, SPR assays tend to be less sensitive, less tolerant to therapeutics, and have lower throughput compared to enzyme-linked immunosorbent assays (ELISAs). In fact, SPR is capable of characterizing early immune responses by detecting and isotyping low-affinity antibodies, which other assays might miss, which makes it much more suitable for immunogenicity assays [70,97]. Furthermore, unlike other immunoassays where the reagents are cost-prohibitive, here the detection equipment is expensive and vendor specific [70,97].

Electrochemiluminescence (ECL) also uses antibodies to bind target impurities. However, unlike the commonly used enzyme-labeled secondary antibodies previously discussed, this technique employs a ruthenium-conjugated protein and tripropylamine (TPA) to produce a detectable, quantifiable luminescent signal. Ruthenium labels are stable, non-radioactive, and offer a choice of convenient coupling chemistries [70]. This is a highly sensitive and selective technique; however, this method requires the production and use of specific antibodies for analyte immobilize and detection, indicating that each impurity must be detected separately [98]. In addition, this technique requires the use of specialized, costly equipment containing carbon electrode plates for detection, which are not necessarily standard in most labs [10,70].

Enzyme-based (EIA) or fluorescence-based (FIA) microtiter plate assays were developed to circumvent the need for method-specific instrumentation and resources experienced with ECL and SPR [10]. This assay involves incubating the sample with a couple of biotinylated antigen-specific antibodies which, after binding and forming immunocomplexes, are removed from solution by association with streptavidin-coated paramagnetic beads. Thereafter, the beads are incubated with enzyme-labeled or fluorescence-labeled antibodies for colorimetric development. By substituting the paramagnetic beads for a solid-substrate surface, the traditional EIA/FIA was transformed into the enzyme-linked immunosorbent assay (ELISA), the most practically useful and commonly employed immunoassay [21]. As previously described, this type of assay employs a series of antibodies to capture specific target antigens. The bound antigen is then complexed with a secondary antibody modified to undergo an enzymatic reaction (colorimetric, fluorescent, or luminescent) for detection via spectrophotometer [70]. However, unlike the EIA/FIA, the use of a solid-substrate surface enables the assay to be set up in various configurations (e.g., sandwich, indirect, bridging, competitive, etc.) for optimal IIMI detection and quantification. ELISAs are relatively sensitive with a detection range of 12–200 ng/mL [10,99]; modern ELISAs have been optimized to improve their sensitivity and allow the detection of analytes at lower (e.g., pg/mL) levels. ELISAs also have high specificity due to their use of analyte-specific antibodies and can be performed relatively quickly (completed in one day) [10,21]. However, the dependency on highly specific antibodies also means that each analyte must be known and analyzed individually, which can be cost-prohibitive [70]. Common HCPs detected via ELISA include anaphylatoxins such as complement C3a [100]; inflammatory cytokines such as IL-1β, IL-6, IL-8, and TNFα [2,101]; and other IIMIs including HMGB1 and flagellin. 

Due to antibody specificity combined with the progression of fluorophore technology, a large number of biomolecules can now be captured from the same small (μL to mL) sample and then detected simultaneously [3,71]. These “multiplex” assays are usually modified ELISA assays, though the EIA/FIA assay format can similarly be multiplexed, as is often used in flow cytometry [3]. Each analyte is then tagged with either a different fluorescent label or organized in a known array for detection via spectrophotometer. As the basic principles of the assay are unchanged, the sensitivity and specificity are still high, though fluorescence bleed-through increases as the number of analytes and fluorophores with similar excitation/emission spectrums increases. In addition, multiplexed assays are less time consuming and labor intensive, while providing higher throughput analysis, compared to an individual ELISA [3].

### 7.5. Nucleic Acid Hybridization Methods

For the detection of nucleic acids in pharmaceutical samples, hybridization techniques such as the dot blot or immunoligand assay (ILA) are often used. The ILA (a.k.a “Threshold Assay”) reliably detects very small amounts of DNA and impurities in liquid solution [102]. This assay employs a biotinylated single-stranded binding (SSB) protein and general anti-ssDNA antibody to complex with any host ssDNA available in the sample. Streptavidin filtration then captures any biotinylated complexes on a specialized matrix-embedded silicon chip, after which the DNA is quantified via enzymatic hydrolysis and subsequent light-addressable potentiometric sensor (LAPS) detection [99]. This method has been shown to be 10–100 times more sensitive than traditional colorimetric or ELISA assays, with a detection range of 5–40 ng/mL [99], requires only small amounts of sample, removes steric binding or stability issues inherent in solid-phase systems, and comes in two formats (sandwich or competitive) depending on the size of the analyte being detected [102] though optimal ssDNA fragments tend to be larger than 600 base pairs [99]. However, this method has reduced specificity due to its sequence-independent binding by general ssDNA antibodies. Furthermore, this technique can be expensive as it requires the use of proprietary equipment, software, and consumables (e.g., silicon chips, specialized buffers, etc.) for quantification [10]. On the other hand, the dot blot employs a substrate covered with immobilized “randomly primed” DNA probes from a known microbial source tagged with radio or fluorescent labels. The probes are exposed to the drug sample allowing for binding between host-cell DNA present in the sample and the specific DNA probes. This binding is then detected and quantified to 3–800 pg/mL against a calibration curve by phosphor- or fluorescence-imaging systems [99]. 

The more popular method of detecting and identifying bacterial and viral nucleic acids is through reverse transcriptase (RT) and quantitative polymerase chain reaction (qPCR) assays [10]. For these assays, trace amounts of DNA or RNA are collected and then amplified through the PCR or RT-PCR method respectively, resulting in many identical copies of the target DNA. The levels of target DNA are then quantified and nucleic acid concentration in the original sample is derived from target copy numbers [99]. Innate immune activation can similarly be assessed by quantifying the levels of pro-inflammatory cytokines, such as IFNγ, IL-1β, IL-6, or other downstream biomarkers by quantifying the levels of target mRNA, amplified as cDNA, which are compared to standard housekeeper genes such as GAPDH or 18S [9,69,103] to determine the fold increase or decrease of the target genes [9,69,103]. Since this process uses specific DNA primers for PCR amplification, the resultant quantification is highly sensitive and specific for the target sequence [99]. However, this also means that species-specific primers must be known. Additionally, as amplification of each nucleic acid fragment requires its own primers, these reactions need to be carried out separately; though, like the previously discussed, multiplexing and proteomics analyses coupled with improvements in high-throughput capabilities have produced arrays of many immobilized primers used to amplify, identify, and quantify many different DNA sequences at the same time [9,21]. This standardization increases the amount of data produced while reducing the required time and labor of these assays [76].

### 7.6. Cell-Based Methods

Since the long-term goal of these studies is the prevention of patient immunotoxicity and possible immunogenicity, more recent assays focus on the in vitro and in vivo impact of IIMIs. These cellular assays detect immune cell activation and proliferation or quantify levels of secreted innate immunity biomarkers (e.g., cytokines, prostaglandins, complement), which may contribute to the process of immunogenicity by priming the immune cells.

Cellular proliferation assays examine the activation and proliferation of specific immune cell subsets, usually, macrophages, neutrophils, or lymphocytes, when treated with the biotherapeutic, compared to control cells and the potential adjuvant effect of known IIMIs [31]. For example, T-cells are activated by concanavalin A or phytohemagglutinin, while B-cells proliferate in response to LPS. While it has long been established that immune cell proliferation in vitro is correlated with cell-mediated immunity, these assays have not been extensively standardized and validated [36]. In addition to needing a skilled technician and the appropriate facilities to support these studies, this assay is time prohibitive as culturing these cells takes at least 48–72 h [36]. 

For a more specific way to determine the type of IIMIs present in a drug formulation, a model of HEK-BLUE cells containing a secreted embryonic alkaline phosphatase (SEAP) reporter inducible by NFκB, transfected with individual TLR receptors, can be used. When bound with their specific agonists alone or in mixtures of IIMIs, the observed NFκB activation for each TLR can be quantified through a colorimetric change. This reporter system has high sensitivity and specificity, similar to what was observed in normal human PBMCs [69,88]. Since therapeutic biologics could mask or interfere with the response of these cell lines, this model necessitates the use of additional inhibition controls. In addition, while this model is effective for detecting TLR-specific IIMIs, it does not yet cover innate immune responses that can be triggered solely through alternate pathways such as CLRs, NLRs, and RLRs. As such, the reporter cells were modified to contain different reporter systems (SEAP, THP-1, and MM6) that would be expressed in the presence of NFκB, TNFα, and mRNA from IL-6 or IL-8 respectively, thereby covering the activation of multiple innate immune responses [69].

Other in vitro models instead directly quantify the levels of cell-secreted immune modulators, such as cytokines and complement proteins (e.g., C3a, C5a), or antibodies [3]. While all of these soluble mediators play an integral role in host defense against microbial invasion, the network of cytokine interactions is responsible for maintaining cellular homeostasis, making them a popular biomarker for gauging the potential immunotoxicity and immunogenicity of new biotherapeutic compounds, especially when compared to normal (untreated) controls [3,36]. Increased levels of cytokines after application of a new drug product can therefore be associated with a product’s immunotoxic effects (either stimulatory or inhibitory), which can lead to adverse patient reactions and reduced therapeutic efficacy due to the formation of ADAs [3]. As such, pharmaceutical immunogenicity is often determined through the use of commercially available multiplexed ELISA assays, chosen based on convenience, affordability, and availability [3], which typically quantify a limited panel of pro-inflammatory cytokines (IL-1, IL-6, or TNFα) [2,87] or subsequent T-effector (T_H_1/T_H_2) cytokines, including IL-2, IL-12, IFNγ or IL-4, IL-5, and IL-6 respectively, even though this may bias analysis towards specific immune pathways [2,3]. Despite the sensitivity and specificity of the multiplex ELISAs used for these analyses, the pleiotropic nature of cytokines and their overlapping activation pathways on numerous target cells [36] often make the results difficult to interpret. Hence, there is a lack of consensus as to which cytokines should be measured to accurately characterize the immunological effects of a new drug. 

### 7.7. In Vivo Methods

A more recent study performed by Haile *et al.* employed an in vivo macaque skin model to better characterize the relationship between type and dose of IIMIs, patterns of innate immune receptors, and pathways triggered by these impurities, and immunogenicity. This model was developed due to the similarity of macaque PBMCs to human PBMCs, and to increase sensitivity compared to traditional murine models which are known to have less sensitive immune cells than those of humans [31]. These studies used mRNA collected after application of known IIMIs, as a basis for comparison to Rasburicase, as a model therapeutic, and measured by qRT-PCR to track the expression of 48 genes involved in the innate immune response, including ILs, TNFs, CD40, GAPDH, etc. [31]. This study demonstrated that, while an increased innate immune response is dependent upon the dose of IIMI administered, the presence of these impurities acted as an adjuvant during co-administration with a protein therapeutic, thereby increasing its immunogenicity. However, it was noted that even trace amounts of IIMIs triggered the transcription of multiple innate immunity genes, emphasizing the need to assess biotherapeutics for a wide variety of possible contaminants and related downstream biomarkers in a more thorough, relevant model, rather than just quantifying levels of specific IIMIs [31]. While the use of animal models is cost-, labor-, and time-prohibitive, these models can provide more applicable data as to the immunotoxicity and immunogenicity of biotherapeutics in humans. 

Overall, methods of cell growth and stimulation are more or less optimized and standardized, and cell-based assays (both in vitro and in vivo) provide the most relevant data on IIMI and drug interactions with the immune system [3,76]. However, they are labor intensive, and the evaluation of cell-secreted biomarkers is subjective due to the cross-reactivity of most immunological pathways and the potential confounding influence of other substances that may modulate the activity of the target substance [3,76].

## 8. Conclusions and Future Directions

It is well documented that the presence of IIMIs in a biotherapeutic formulation can trigger immunotoxicity and, with repeated exposure, immunogenicity against the therapeutic [31,80,81,82]. To prevent these adverse patient reactions, the FDA currently requires quantification of five key IIMIs: LPS, HMGB1, β-glucan, flagellin, and nucleic acids [62,69], to demonstrate biotherapeutic safety, quality, and clinical performance. These guidelines aim to mitigate the formation of future ADAs through commonly activated innate immune receptors, specifically TLRs, CLRs, and complement. However, these guidelines do not necessarily account for potential immunotoxic responses to other IIMIs that may be present in the formulation. As such, the FDA panel of IIMIs required for quantification should be expanded to cover a much broader repertoire of impurities, including microbial antigens that can potentially trigger other innate immunity pathways, common manufacturing leachates, and solvents, and toxic additives required for maintaining host cells. The list of possible leachates, solvents, and host cell additives will be extensive, requiring tailoring to the specific processes employed during manufacturing and purification [62]. As for the microbial IIMIs, most innate immunity receptors and pathways can be covered using ten common IIMIs, some of which are already required and discussed, including flagellin, FSL-1, zymosan, ODN2006, and ODN2216, both high- and low-molecular-weight poly(I:C), MDP, CLO75, and LPS. While this ten IIMI panel necessitates more laboratory testing before new drugs can gain approval, adhering to the ppm levels for these required IIMIs will demonstrate that little/no immunotoxicity will result from trace levels of substances present in the drug formulation, therefore reducing the potential for immunogenicity. 

Second, to measure and quantify IIMIs present in biotherapeutic formulations, a variety of available assays have been discussed. As genomic and proteomic technology advances, these assays have become more sensitive and specific, enabling improved detection and quantification of IIMIs. In addition, many of these assays are now being coupled into high-throughput formats which can produce more data with reduced sample and reagent volumes, as well as cost and labor expenditures. However, due to the variety of potential IIMIs, there is currently no single assay that can provide a profile of all IIMIs present within a biotherapeutic [70]. Moreover, there is a lack of agreement as to which analytical assays should be standardized [21] so most studies use a series of assays to broadly cover the detection of all possible IIMIs present in biopharmaceuticals [21,71]. To better standardize results across experiments and laboratories, the use of a single high-throughput platform capable of detecting a wide panel of biomarkers of the same class (small molecules, proteins, or nucleic acids) in parallel, such as multiplexed ELISAs, MS, or genomic arrays, should be employed. 

Finally, given that immunostimulation is the overall concern, the use of newer cell-based assays which track levels of biomarkers (e.g., cytokines, transcription factors, mRNA [62]) affected by the presence of IIMIs, rather than the individual IIMIs themselves, can provide a stronger connection between the applied biotherapeutic and its impact on immunotoxicity and immunogenicity [31]. Past cellular studies focusing on a limited selection of cytokines and chemokines, usually, a combination of pro-inflammatory IL-1, IL-8, IL-6, TNFs, and IFNs, have failed to adequately interrogate the entire immune cascade [2,36]. Since immunotoxicity can cover a range of patient responses from immunostimulation and HSR to immunosuppression, measuring a wider assortment of cytokines, including but not limited to IFNs (α, γ, λ); ILs (1α/β, 2, 6, 8, 10, 12, 17); interferon-gamma inducible protein (IP-10); TNFα, prostaglandin-E_2_ (PGE-2), macrophage inflammatory protein (MIP-1α), and monocyte chemoattractant protein (MCP-1), can provide a more complete picture as to the type and degree of immunotoxic response that can potentially be triggered by a new biotherapeutic formulation.

## Figures and Tables

**Table 1:** Examples of guidance documents and international standards for the measurement of impurities in therapeutic products. International standards (IS) and Guidance for Industry (GI) provided through the U.S. FDA, the U.S. Pharmacopeia (USP), and the International Organization for Standardization (ISO) describe the risks of endotoxin and pyrogen contamination in therapeutic products and outline the assays, protocols, and detection limits which have been standardized and approved for universal application in therapeutic safety and purity measurements.

Document	Type	Purpose	Reference
USP 85 Bacterial Endotoxins Test	GI	Describes method validation and sample preparation requirements for turbidity, chromogenic and gel-clot LAL assay	[63]
USP 151 Pyrogen Test	GI	Describes method validation and sample preparation requirements for the rabbit pyrogen test	[64]
FDA Immunotoxicity Testing Guidance (FDA-modified version of ISO-10993)	GI	Summarizes general types of toxicity and subsequent testing that should be considered for medical devices or constituent materials	[35]
FDA Guidance for Industry: Pyrogen and Endotoxin Testing: Questions and Answers	GI	Provides bacterial endotoxin and pyrogen testing recommendations (gel-blot, photometric, and kinetic tests) and acceptance criteria	[65]
FDA Endotoxin Testing Recommendations for Single-Use Intraocular Ophthalmic Devices	GI	Provides recommended endotoxin limits for the release of intraocular devices and single-use intraocular ophthalmic surgical instruments/accessories	[66]
FDA Questions and Answers on Quality Related Controlled Correspondence	GI	Provides answers to common scientific and regulatory questions around the manufacture and quality control of generic drug production including endotoxin testing	[67]
FDA Immunogenicity Assessment for Therapeutic Protein Products	GI	Outlines approaches to evaluate and mitigate adverse immune responses/immunogenicity associated with therapeutic protein products; discusses the importance of IIMI detection	[62]
ISO-10993-1 Biological Evaluation and Testing Standards for Medical Devices (prepared by ISO/TC 149)	IS	Outlines the potential biological risks arising from the use of medical devices and provides a framework to plan biological evaluation, testing methods, and acceptance criteria	[68]
ISO-29701 Endotoxin Standard (prepared by ISO/TC 229)	IS	Describes application of LAL assay for evaluation of endotoxin levels in nanomaterials intended for use in vitro	[29]
ISO-21582 Pyrogenicity Standard (prepared by ISO/TC 149)	IS	Specifies the principles and methods for pyrogen testing of medical devices and their materials	[72]
